# Wet-spinning of magneto-responsive helical chitosan microfibers

**DOI:** 10.3762/bjnano.11.83

**Published:** 2020-07-07

**Authors:** Dorothea Brüggemann, Johanna Michel, Naiana Suter, Matheus Grande de Aguiar, Michael Maas

**Affiliations:** 1 Institute for Biophysics, University of Bremen, Otto-Hahn-Allee 1, 28359 Bremen, Germany; 2 MAPEX Center for Materials and Processes, University of Bremen, 28359 Bremen, Germany; 3 Department of Biomimetics, Hochschule Bremen - City University of Applied Sciences, Neustadtswall 30, 28199 Bremen, Germany; 4 Advanced Ceramics, University of Bremen, Am Biologischen Garten 2, 28359 Bremen, Germany

**Keywords:** biocompatible actuators, chitosan fibers, helical fibers, magnetic tissue engineering, mechanical properties, wet-spinning

## Abstract

Helical structures can be found in nature at various length scales ranging from the molecular level to the macroscale. Due to their ability to store mechanical energy and to optimize the accessible surface area, helical shapes contribute particularly to motion-driven processes and structural reinforcement. Due to these special features, helical fibers have become highly attractive for biotechnological and tissue engineering applications. However, there are only a few methods available for the production of biocompatible helical microfibers. Given that, we present here a simple technique for the fabrication of helical chitosan microfibers with embedded magnetic nanoparticles. Composite fibers were prepared by wet-spinning and coagulation in an ethanol bath. Thereby, no toxic components were introduced into the wet-spun chitosan fibers. After drying, the helical fibers had a diameter of approximately 130 µm. Scanning electron microscopy analysis of wet-spun helices revealed that the magnetic nanoparticles agglomerated into clusters inside the fiber matrix. The helical constructs exhibited a diameter of approximately 500 µm with one to two windings per millimeter. Due to their ferromagnetic properties they are easily attracted to a permanent magnet. The results from the tensile testing show that the helical chitosan microfibers exhibited an average Young’s modulus of 14 MPa. By taking advantage of the magnetic properties of the feedstock solution, the production of the helical fibers could be automated. The fabrication of the helical fibers was achieved by utilizing the magnetic properties of the feedstock solution and winding the emerging fiber around a rotating magnetic collector needle upon coagulation. In summary, our helical chitosan microfibers are very attractive for future use in magnetic tissue engineering or for the development of biocompatible actuator systems.

## Introduction

Helical fibrous structures are ubiquitous in nature and are found at virtually every length scale. A few examples are the structural motifs in proteins and DNA at the molecular level [1], the nanoscopic flagella in bacteria [2], the spiral shape of some bacteria (e.g., *Helicobacter pylori*) [3], the chiral seed pods [4], and the macroscopic tendrils of climbing plants [5–6]. With their ability to store mechanical energy and to optimize the accessible surface area of a biological material, helical structures mainly serve two purposes in nature: structural reinforcement and motion [5,7]. Despite the biological relevance of helical structures, to date very few strategies have been developed to fabricate biocompatible helical fibers on the microscale. Such helical assemblies have high applicability in biology and have been largely used as actuators for micro- or nanoswimmers during gene, drug or chemical delivery, as scaffolding for microtissue constructs, or as a tool to study the fundamental aspects of biological motion and structure formation [8–12]. Beyond the usual requirements for tissue engineering materials (i.e., full biocompatibility, integration, and stimulation of the respective tissues), motion-activated cell support should also exhibit mechanical compliance with the surrounding tissue environment [13–15].

Natural polymers, in particular polysaccharides such as alginate, hyaluronic acid or chitosan, are widely used as biocompatible materials since they are biochemically similar to the native extracellular matrix (ECM) [16]. Chitosan is a biopolymer that combines excellent biocompatibility, low toxicity and antibacterial properties with a low immunogenicity [17] and, therefore, has become highly used in tissue regeneration [18–19]. Chitosan fibers are particularly well-suited for tissue engineering due to their highly porous scaffold architecture [20]. Using electrospinning, chitosan fibers can be produced with a diameter ranging from several tens of nanometers to a few micrometers [21]. Blends of chitosan with alginate, silk, fibroin, cellulose or collagen can also be processed into composite fibers by electrospinning [22]. Wet-spinning is another well-established method of fabricating chitosan fibers with a diameter in the micrometer range [23–24]. Since cells are also on the same length scale, these fibers can be used as soft biological actuators to mechanically stimulate cell growth [25]. During wet-spinning, chitosan is extruded through thin needles from acidic solutions into a coagulation bath [24]. Typical coagulants used are acetone [26–27], alcohols [28–29] or alkaline solvents [30–32]. Regarding future tissue engineering applications, wet-spun chitosan fibers have already been found to serve as scaffolds for osteoblasts [20], Schwann cells [33], hepatoma HepG2 cells [34] and porcine valvular interstitial cells [35].

Additionally, by using either wet-spinning or electrospinning techniques, nanoparticles can be suspended into the viscous spinning solution and embedded into the fiber matrix. For example, silver nanoparticles have been incorporated into electrospun chitosan fibers enabling antibacterial activity in wound dressings [36], and nanohydroxyapatite was embedded into chitosan fibers for bone tissue engineering applications [37]. Likewise, magnetic iron oxide particles have been blended with chitosan to prepare electrospun composite fibers [38–39] to form magneto-responsive polymer nanocomposites for bone tissue engineering [40] or to facilitate 3D printing of magnetized chitosan solutions into helical microswimmers for drug delivery [11]. In the future, the incorporation of magnetic particles into biocompatible fibers might pave the way for the development of new soft biological motors, which can mechanically steer cell behavior in vivo by applying external stimuli [41–42]. Emerging fields, such as magnetic tissue engineering, which uses magnetic levitation to control cell growth, would greatly benefit from the use of magnetic scaffolds since these would replace the need for treating the cells with magnetic iron oxide nanoparticles in order to generate 3D spheroids [43–46].

The production of helical microfibers can, in principle, be achieved by adding a rotation element to the translational extrusion process. This has been demonstrated by Sun et al. who developed a magnetic micromanipulation technique in which freshly-spun alginate fibers containing magnetic nanoparticles were fixed with a magnetized conical tip and rotated around micropillars, acquiring a helical shape with three to four windings [47]. Non-scalable procedures, such as high-temperature synthesis, photolithography or the use of organic templates are examples of alternative ways to synthesize helical nano- or microfibers from various materials like carbon nanotubes (CNTs), ZnO or different polymers [8,48–49].

Here, we present a simple method for synthesizing helical chitosan microfibers with embedded magnetic nanoparticles. This strategy is based on wet-spinning of magnetic fibers, which are collected on a rotating needle controlled by an external magnetic field with a predefined helical geometry. The fibers were characterized regarding their morphology, microstructure, magnetization and mechanical characteristics. These helical fibers have the potential to be used as novel actuator systems or as magneto-responsive scaffolds for tissue engineering.

## Results and Discussion

**Figure 1:**
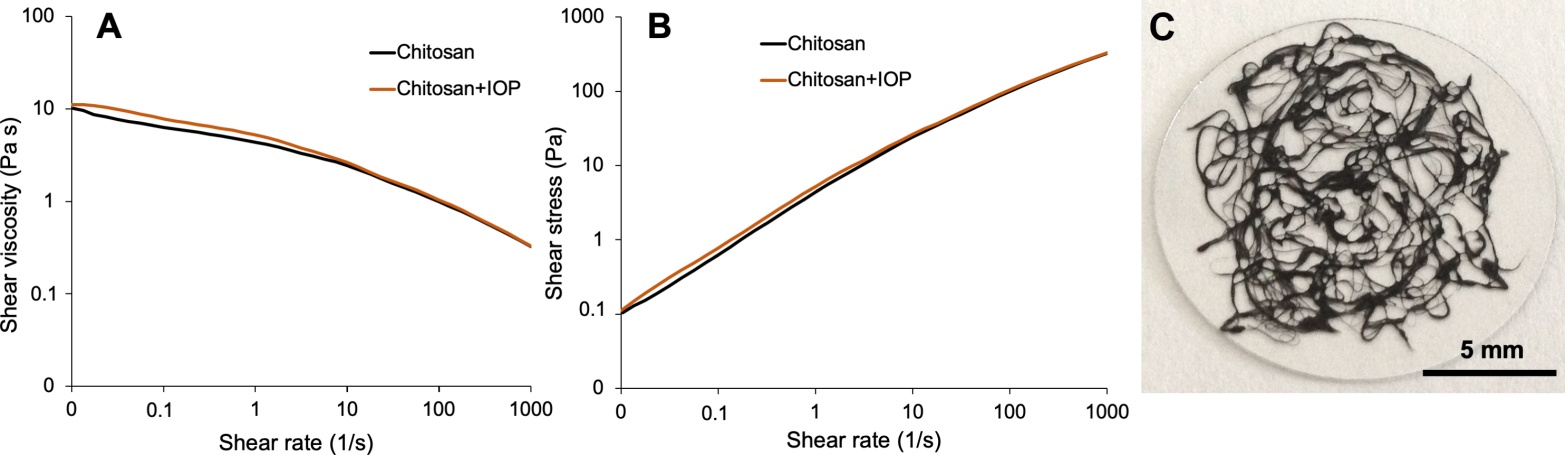
(A, B) Shear viscosity and shear stress of bare chitosan solutions (black lines) and chitosan solutions containing IOPs (orange lines). The precursor solution consisted of 30 mg·mL^−1^ chitosan and 10 mg·mL^−1^ IOPs. (C) Chitosan–IOP microfibers on a glass slide obtained by wet-spinning followed by coagulation in ethanol.

The viscous feedstock solutions containing 30 mg·mL^−1^ chitosan and 10 mg·mL^−1^ magnetic iron oxide particles (IOPs) showed a pronounced shear-thinning behavior. These results were corroborated by previous studies that used aqueous chitosan solutions for wet-spinning experiments [28,50]. A zero-shear viscosity of approximately 10 Pa·s (Figure 1A) was obtained. The yield point was not strongly pronounced at 0.1 Pa (Figure 1B), which explains why further solidification of the fiber matrix was necessary to form stable fibers.

The magnetic properties of wet-spun fibers can be tuned by the amount of magnetic IOPs within these fibers. Therefore, controlling the IOP concentration is critical for magnetically reeling these fibers into a helical construct. Therefore, we initially used different blend solutions prepared using an IOP concentration range varying between 1 and 10 mg·mL^−1^. This is the same concentration range previously used for 3D-printing chitosan helices [11]. Microfibers were then prepared by wet-spinning in our self-built wet-spinning setup, which was comprised of a syringe pump and an ethanol coagulation bath (Figure S1, Supporting Information File 1). The most stable and reproducible wet-spinning conditions were obtained at an IOP concentration of 10 mg·mL^−1^ in 30 mg·mL^−1^ chitosan solution. Under these conditions, IOP-embedded chitosan microfibers were prepared in a reproducible manner, which could be recognized by the black color of the fibers (Figure 1C). Due to the high reproducibility of the generated wet-spun microfibers, the 10 mg·mL^−1^ IOP concentration was also used for manufacturing the helical constructs. Evidence showed that only disordered fiber mats were observed after being retrieved from the ethanol bath. These results validate wet-spinning as a technique that can be used to embed nanoparticles into chitosan fibers. Until now, this achievement has only been reported in electrospinning [36–38] or 3D-bioprinting [11] techniques.

**Figure 2:**
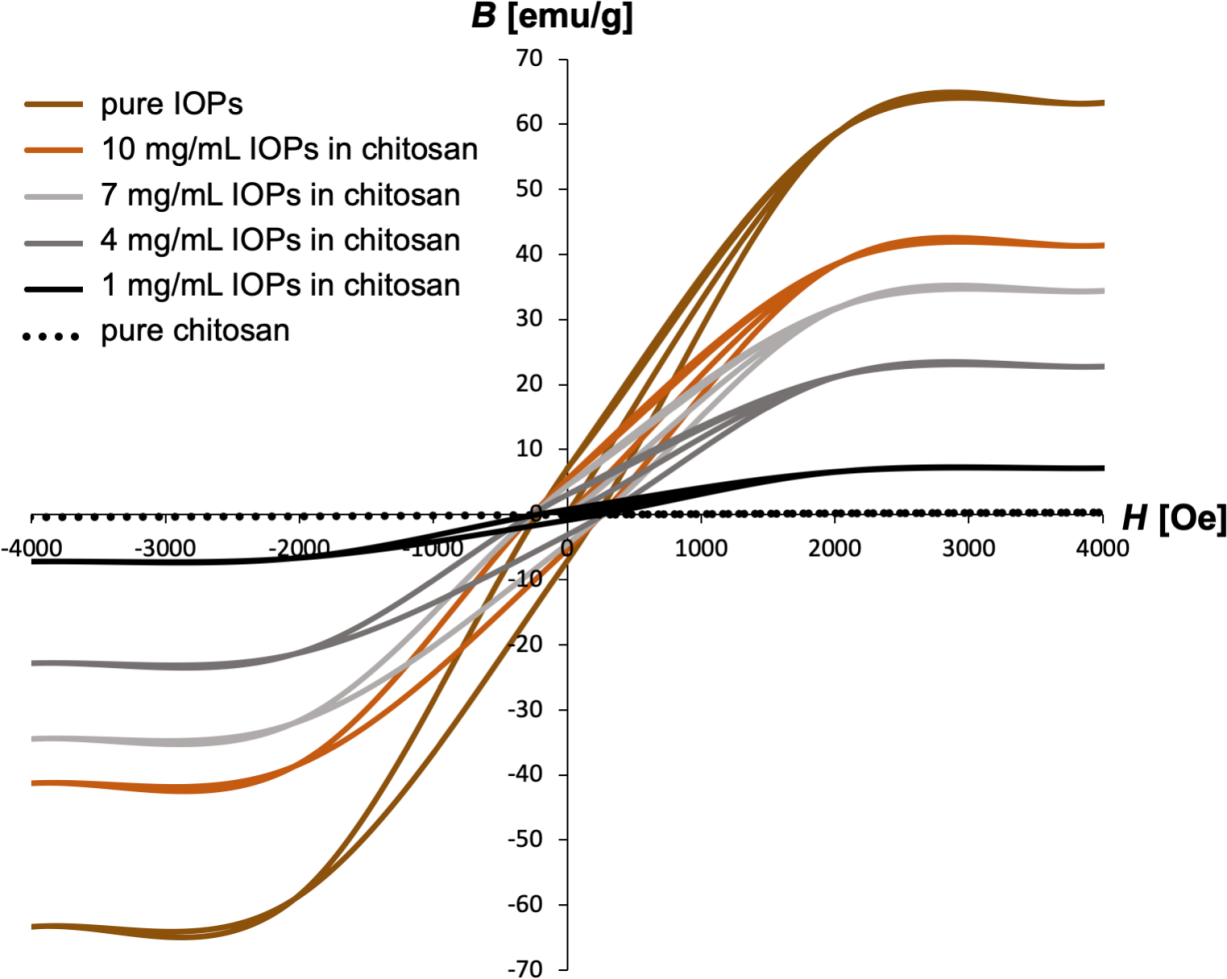
Vibrating sample magnetometer analysis of bare IOPs (brown line), bare chitosan fibers (dashed black line) and chitosan microfibers containing different iron oxide nanoparticle concentrations (10 mg·mL^−1^ IOP: orange line, 7 mg·mL^−1^ IOP: light gray line, 4 mg·mL^−1^ IOP: dark gray line, 1 mg·mL^−1^ IOP: black line). The magnetic saturation of the composite fibers increased with an increase in nanoparticle concentration.

Vibrating-sample magnetometer (VSM) analysis was used to characterize the magnetic properties of the wet-spun IOP-embedded chitosan fiber networks (Figure 1C). As expected, the sample magnetization could be controlled by the IOP concentration (Figure 2). At 10 mg·mL^−1^ (the maximum IOP concentration used) the fibers showed a fairly strong magnetic saturation at 40 emu·g^−1^. This value is higher than what had been previously reported for chitosan-based fiber blends containing magnetic nanoparticles with a diameter varying between 10 and 30 nm [38] or in another study with a diameter of 5.3 nm [39]. Since the magnetic particles used in this work were approximately 100 nm in diameter, the wet-spun chitosan fibers obtained were clearly ferromagnetic rather than superparamagnetic [40]. This observation was confirmed by the magnetic hysteresis curves which showed a maximum remanent magnetization value of 7.69 emu·g^−1^ (see Table S1 in Supporting Information File 1 for further data on the magnetic properties of the samples). Although the magnetization was only analyzed for dried fibers, a similar magnetic behavior is expected for the viscous feed solution. This effect was subsequently used to optimize the collection and winding of the fibers during the manufacturing of helical chitosan structures.

To facilitate the characterization of helical chitosan microfibers, wet-spun fibers were harvested from the coagulation bath, manually wound up on a magnetized needle and left for drying. After this process, helical IOP-embedded chitosan fibers (≈130 µm in diameter) were reproducibly obtained. This diameter was within the same range that was previously reported for wet-spun chitosan–alginate fibers [51]. In contrast, wet-spinning of pure chitosan solutions yielded smaller microfibers (*d* = 10 to 20 µm) [23–24]. For future applications in the bioactuator field, key parameters in the wet-spinning process, such as the flow speed or the IOP concentration, might need to be adjusted in order to further miniaturize the helical chitosan fibers.

**Figure 3:**
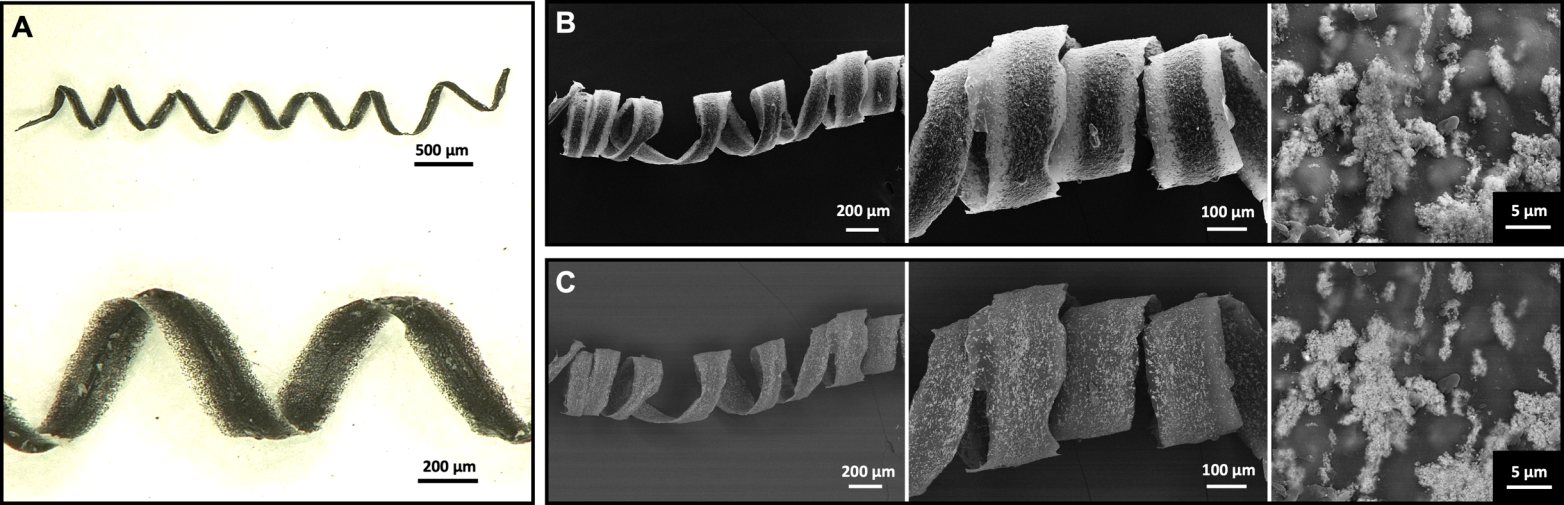
Helical IOP-embedded chitosan microfibers were prepared by wet-spinning and manual winding. (A) A representative light microscopy image shows that the helical fibers have a flat surface. (B) SEM images obtained from the secondary electron detector (left and middle panel) reveal a fiber diameter of approximately 130 µm and a higher magnification image confirms the coagulation-driven IOP agglomeration in the chitosan fibers (right panel). (C) SEM images obtained from the backscattered electrons show the difference in material contrast between the chitosan matrix and embedded IOPs at different magnifications.

The dried chitosan–IOP microfibers exhibited haptic properties comparable to the human hair and the total fiber length was in the range of a few centimeters. When the manual winding was completed, the helical fibers had a diameter of approximately 500 µm at one to two windings per millimeter of fiber length and the primary fibers (*d* = 130 µm) were flat as a result of their soft nature during the winding process (Figure 3A). This appearance resembled the morphology of helical alginate microfibers, where fiber shaping was achieved by micromanipulation in a magnetic field [47]. In comparison to the aforementioned work [47] and also to the 3D-printed chitosan microswimmers containing magnetic nanoparticles (length = 20 µm, outer diameter = 6 µm) [11], the wet-spun helical fibers generated in this work were significantly larger and longer.

The morphological analysis based on SEM images obtained from the wet-spun composite fibers revealed that the IOPs were homogeneously embedded as micrometer-sized agglomerates in the chitosan matrix (Figure 3B,C). Individual IOPs had a diameter of approximately 100 nm whereas their aggregates had a diameter on the order of several micrometers. According to the dynamic light scattering results (Table S2, Supporting Information File 1), small IOP agglomerates were already present in the acidic stock solution. The particle distribution obtained in this work differs from previous studies in which magnetic nanoparticles (diameter between 5 and 12 nm) were either blended into electrospun chitosan-based fibers [38] or loaded onto the fiber surface by a post-treatment [39]. To tailor our wet-spun magnetic chitosan fibers for possible applications in magnetic tissue engineering [44,46] it will be important to determine the effect that smaller magnetic particles have on the microfiber morphology, on the particle distribution and on the overall scaffold magnetization.

**Figure 4:**
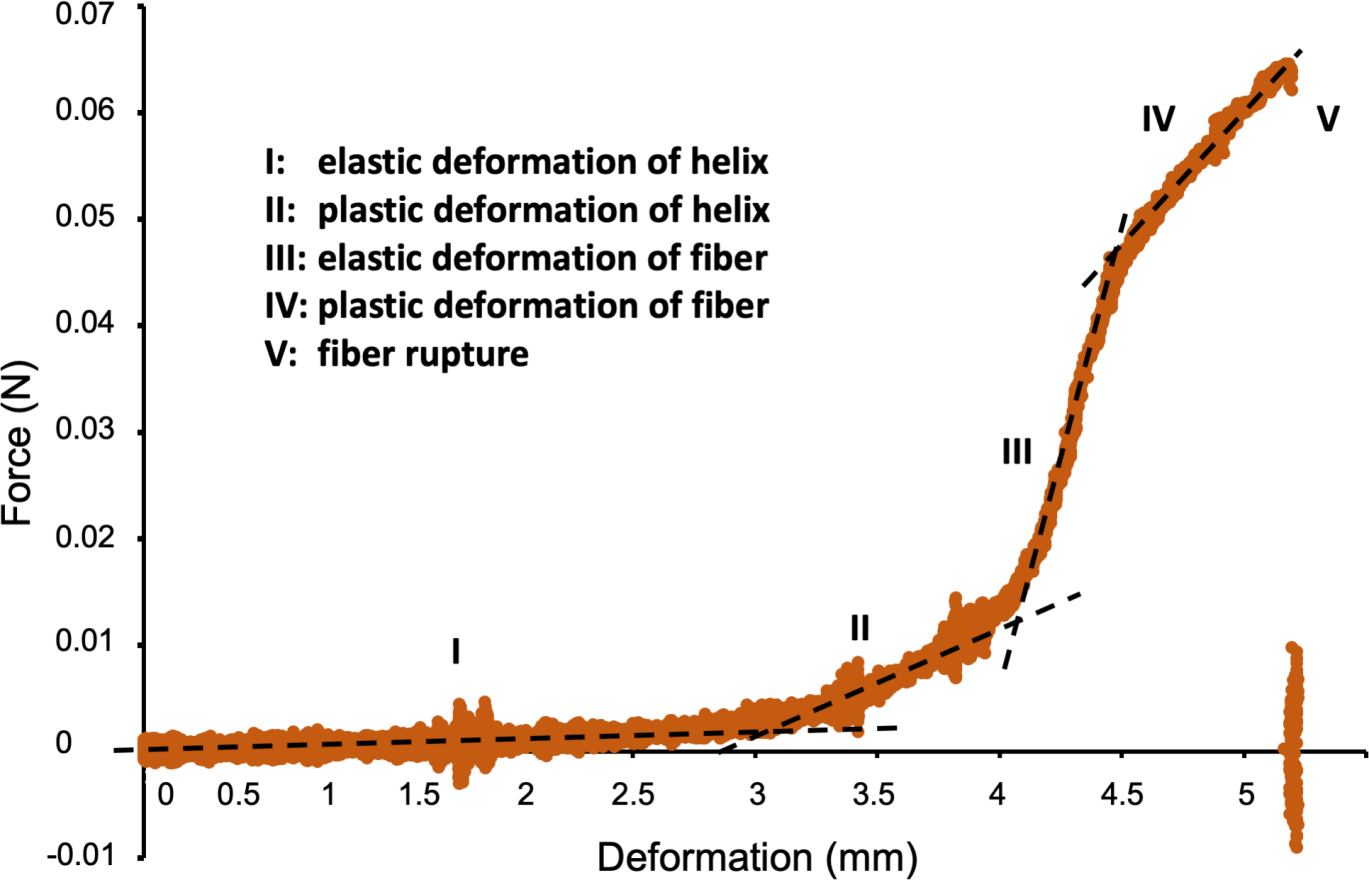
Mechanical characteristics of IOP-embedded helical chitosan fibers showed different deformation regimes until the composite fiber ruptured.

Subsequently, we analyzed the mechanical properties of our manually prepared helical fibers in a customized tensile testing machine, which was adapted for fiber testing under tensile loads. A representative force (N) vs deformation (nm) curve is shown in Figure 4. As expected, at low deformation the helical fibers behaved similarly to springs and deformed elastically (Figure 4, range I). In this elastic regime an average spring constant of 0.3 ± 0.4 N·m^−1^ was found (averaged over 29 fibers with average length of 9 mm). Normalized to a single fiber winding, the average spring constant was 2.7 ± 2.4 N·m^−1^. Upon further stretching and unwinding, the fiber reached the plastic regime until fully stretched (Figure 4, range II). This stretching was then followed by an elastic deformation of the unwound fiber (Figure 4, range III), which transformed to a plastic deformation (Figure 4, range IV) and eventually led to failure when the fiber ruptured (Figure 4, range V). The fibers were reasonably stable during the experiment and had an average Young’s modulus of 14 MPa. In addition, a control experiment was performed in which straight bare chitosan fibers were submitted to mechanical testing. The results revealed that those fibers had a Young’s modulus of 166 MPa which was in the same range as the values obtained for the IOP-embedded helical fibers. This observation shows that an IOP concentration of 10 mg·mL^−1^ did not significantly change the mechanical properties of bare chitosan fibers.

As expected, the mechanical properties of the fibers measured during the elastic regime were not significantly influenced by the presence of the embedded IOPs since our previous rheological characterization revealed that the viscoelastic properties were mainly dominated by the chitosan matrix (Figure 1). In the plastic regime, however, the presence of IOP aggregates might lead to an earlier fracture than expected for bare chitosan helices. Unfortunately, the mechanical characterization of bare chitosan helices was not possible in the current experimental setup since it would require the winding of a magnetized feed solution. To confirm our hypothesis that different IOP concentrations may influence helix rupturing, further experiments, such as in situ SEM characterization of helix fracture, would be necessary.

Similar values of Young’s modulus in the range of 4 to 12 MPa were previously obtained for wet-spinning of bare chitosan solutions into ammonia [35]. These results were in good agreement with the Young’s modulus values obtained for our helical chitosan fibers. On the other hand, gel-spinning in combination with a post-drying step led to much higher Young’s modulus values between 3 and 5 GPa. Interestingly, wet-spun fiber blends of chitosan and alginate with a diameter of approximately 100 µm exhibited a Young’s modulus of approximately 0.8 MPa [51]. Besides post-drying, stretching and chemical cross-linking were other factors that changed the Young’s modulus of chitosan fibers [52–53]. These are important parameters that could be used for controlling the mechanical properties of our magnetic chitosan helices in order to achieve mechanical compliance with the surrounding tissue environment [14].

**Figure 5:**
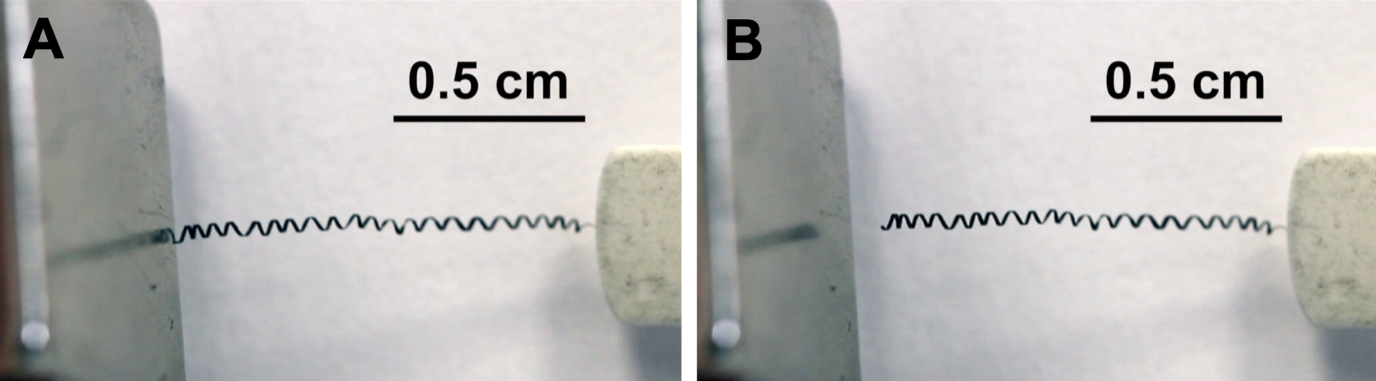
Freeze frames from a video showing attraction to and reversible stretching of a helical fiber by a permanent neodymium magnet (see Supporting Information File 2 for the full video).

In addition, helically shaped chitosan IOP fibers could be easily attracted to and reversibly stretched by a permanent neodymium magnet (Figure 5 and Supporting Information File 2). Based on what has already been established in terms of biocompatibility of chitosan–IOP blends [39], the fibers generated in this study are therefore highly interesting for future use as a magnetic cell scaffold in actuator systems.

**Figure 6:**
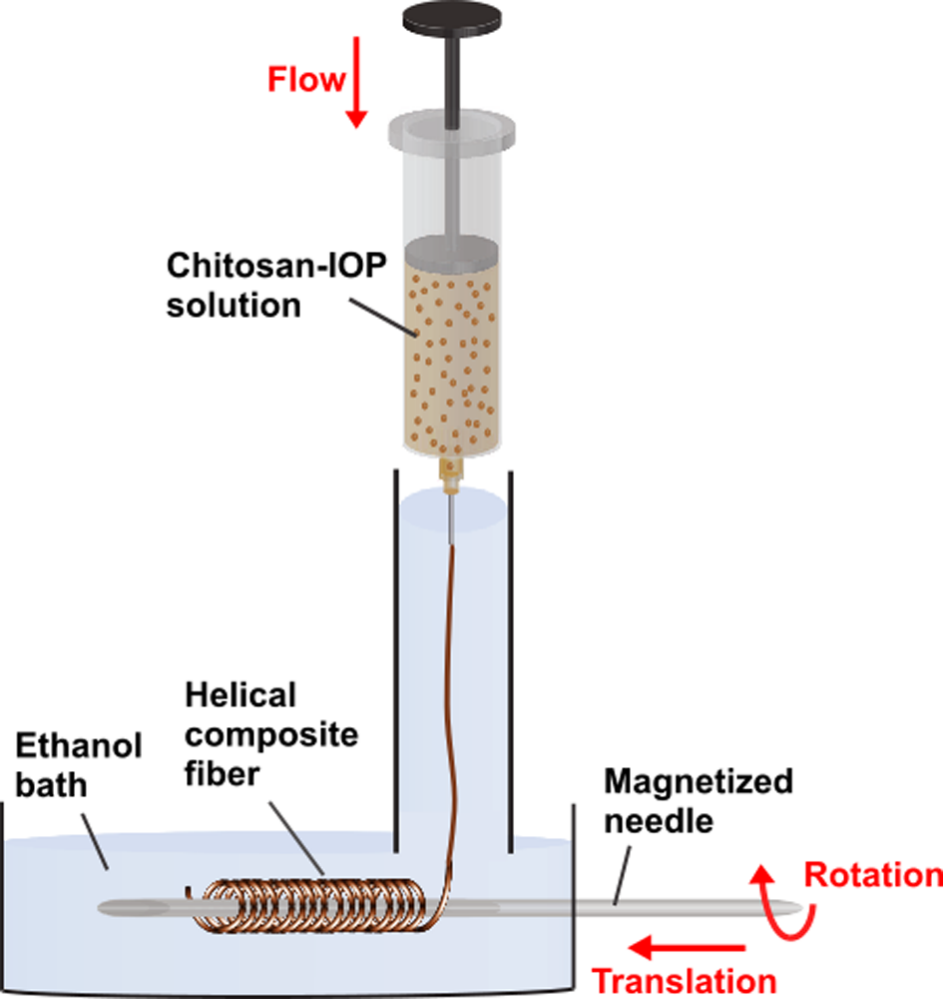
Scheme of the wet-spinning process that generated helical microfibers. A solution of chitosan and magnetic iron oxide nanoparticles was extruded into an ethanol coagulation bath. The emerging fibers were collected by a teflon-coated rotating needle with a stainless steel core, which simultaneously performed a translational movement to achieve a helical fiber shape.

To maintain the reproducibility during the preparation of magnetic helical chitosan fibers we adapted the design of our initial setup and introduced several additional elements (Figure 6) which were automated using a Lego Mindstorms NXT set (Figure S2, Supporting Information File 1). Fiber extrusion into a 50 cm long coagulation tube enabled a quick (≈1 s) initial solidification of the viscous extrudate, which was essential for the subsequent manipulations that led to a helical shape. For collection and winding, we introduced a teflon-coated rotating needle with a stainless steel core at the bottom of the coagulation bath. The needle was placed perpendicular to the direction of the feedstock solution flow to facilitate subsequent fiber winding. For this purpose, a rotational movement of the needle was combined with a simultaneous translational movement perpendicular to the wet-spinning flow. A translational speed of 3.8 mm·s^−1^ and a rotational speed of 1.8 mm·s^−1^ were found to be the optimum conditions to twist chitosan fibers into helical shapes. This rotational speed was 100% higher than that previously used by Sun and co-workers, who processed blends of alginate and magnetic nanoparticles into helical fibers in a microfluidic device [47].

## Conclusion

We introduced a straightforward approach for the production of helical chitosan microfibers with embedded magnetic nanoparticles. Composite fibers were produced by wet-spinning into an ethanol bath and exhibited a diameter of approximately 130 µm upon drying, which agrees well with previous studies on blended chitosan fibers. With our approach we could show that wet-spinning is ideal to embed magnetic nanoparticles into chitosan fibers without introducing any toxic components into the final product. By subsequent winding we produced helical-shaped fibers with a diameter of approximately 500 µm with one to two windings per millimeter. SEM analysis of wet-spun helical fibers revealed that the IOPs were distributed inside the fiber matrix in larger clusters of 100 nm, resulting in strong ferromagnetic properties of the fibers. Since the generated fibers had magnetic properties they could be attracted to and stretched by a permanent magnet. The dried chitosan helices had a spring-like elastic behavior. The composite fibers exhibited a Young’s modulus of approximately 14 MPa, which was in the range of wet-spun bare chitosan fibers. By utilizing the magnetic properties of the blend solution, the winding process could be subsequently automated. Similar to previous works with helical alginate fibers, wet-spinning twisting of the magnetic chitosan fibers led to a flat profile of the latter. Based on our results, magnetic chitosan helices may have high applicability in magnetic tissue engineering as a magnetic and motion-activated cell scaffold.

## Experimental

### Preparation of chitosan solutions with IOPs

Magnetic iron(II,III) oxide nanoparticles (Sigma-Aldrich, Munich, Germany) with a diameter ranging from 50 to 100 nm were suspended in 1% acetic acid (Sigma) using an ultrasonicator (Branson, Danbury, United States) for 10 min. Unless stated otherwise, an IOP concentration of 10 mg·mL^−1^ was used. Low-molecular-weight chitosan with a molar mass between 50,000 and 190,000 g·mol^−1^ (Sigma) was added to the IOPs at a final concentration of 30 mg·mL^−1^. All solutions were prepared with deionized water from a TKA water purification system (Thermo Fisher Scientific, Schwerte, Germany).

Rheological characterization of the aforementioned solutions was carried out on a Kinexus Pro rheometer (Malvern, Herrenberg, Germany) with a cone-plate geometry (1° angle and 50 mm diameter). The shear stress was measured at 20 °C with a stepwise increase in the shear rate and a one minute holding time at each shear-rate step. The size and zeta potential measurements were performed with 10 mg·mL^−1^ IOP dispersions in 1% acetic acid using a dynamic light scattering device (ZetaSizer NanoSP, Malvern, United Kingdom).

### Preparation of chitosan microfibers with IOPs

A coagulation bath containing absolute ethanol (VWR, Darmstadt, Germany) was prepared in a polyethylene terephthalate glycol tube (MOCAP, Telford, UK) for wet-spinning in a custom-built setup (Figure S1, Supporting Information File 1). Injection syringes (Braun, Melsungen, Germany) were filled with a chitosan–IOP solution and mounted on a syringe pump (World Precision Instruments GmbH, Berlin, Germany). Needles with an inner diameter of 0.25 mm and a blunt-shaped tip (smt Sander, Potsdam, Germany) were connected to the syringes through luer lock adapters (Carl Roth, Karlsruhe, Germany) and immersed into the coagulation bath. The wet-spinning of continuous chitosan fibers embedded with IOPs was carried out at a 20 mL·h^−1^ flow rate. After 30 min, the fibers were collected from the coagulation bath and dried overnight under ambient conditions. To facilitate further analysis, the composite fibers were transferred to glass coverslips (VWR, Darmstadt, Germany). For the manual production of helical microfibers, wet-spun extrudates were collected from the coagulation bath and reeled around a 0.3 mm diameter magnetic needle. Subsequently, a full automatization of this process was achieved by using a Lego Mindstorms NXT setup.

### Scanning electron microscopy

Morphological analysis of the IOP-embedded chitosan fibers was performed under a scanning electron microscope (SEM) using a Zeiss Supra 40 field-emission device (Carl Zeiss, Oberkochen, Germany) at an acceleration voltage of 3 kV. Prior to SEM analysis, dried fibers were sputter-coated with a 7 nm gold layer using a Bal-Tec SCD 005 sputter system (Leica Microsystems, Wetzlar, Germany).

### Vibrating sample magnetometer analysis

An EZ9 vibrating sample magnetometer (Microsense, Rottweil, Germany) was used to analyze the magnetization of chitosan fibers with varying IOP concentration. Prior to magnetometer analysis, the fibers were dried under ambient conditions for 24 h. Hysteresis curves were obtained at a magnetic field strength of 40 × 10^3^ Gs using 72 points per loop with a scan speed of 10 s per point. The range covered during the hysteresis scan was between +22 × 10^3^ Oe and −22 × 10^3^ Oe. During the scans, the magnetic field was measured with a FCM-10 control module and the magnetization data were analyzed using the EasyVSM software, both embedded into the EZ9 device.

### Mechanical characterization of helical fibers

Single filament tensile tests were performed according to the norms DIN EN 1007-4 and 1007-6. The fibers were tested in a self-made tensile testing machine equipped with a 1 N load cell, a ULC-1N-535 model interface, and a ±1 mm length linear variable differential transformer (LVDT) sensor. System compliance was measured prior to testing. Twenty nine samples were tested under a travelling speed of 1 mm/min until failure. Before testing, the cross-sectional area of every fiber was measured in an optical microscope (SENSOFAR PLl 2300, Nikon, Tokyo, Japan). Thus, the stress–strain relation of each individual fiber could be determined by taking into account the gauge length of 25 mm.

## Supporting Information

File 1 Additional experimental data.

File 2 Video showing the magnetic manipulation of a helical chitosan fiber.
Magnetic manipulation of a wet-spun helical chitosan fiber with embedded IOPs using a neodymium magnet.
