# Supplementary material for: Wet-spinning of magneto-responsive helical chitosan microfibers
Source: Beilstein J Nanotechnol. 2020 Jul 7;11:991–9. doi: 10.3762/bjnano.11.83 (PMC7356321; doi:10.3762/bjnano.11.83)
Supplement: File 1 — Additional experimental data. [file Beilstein_J_Nanotechnol-11-991-s001.pdf]

BEILSTEIN JOURNAL OF NANOTECHNOLOGY

Supporting Information

for

Wet-spinning of magneto-responsive helical chitosan microfibers

Dorothea Brüggemann, Johanna Michel, Naiana Suter, Matheus Grande de Aguiar and Michael Maas

Beilstein J. Nanotechnol. **2020,** *11,* 991–999. doi:10.3762/bjnano.11.83

Additional experimental data

License and Terms: This is a supporting information file under the terms of the Creative Commons Attribution License (http://creativecommons.org/licenses/by/4.0). Please note that the reuse, redistribution and reproduction in particular requires that the authors and source are credited.
The license is subject to the *Beilstein Journal of Nanotechnology* terms and conditions: (https://www.beilstein-journals.org/bjnano)

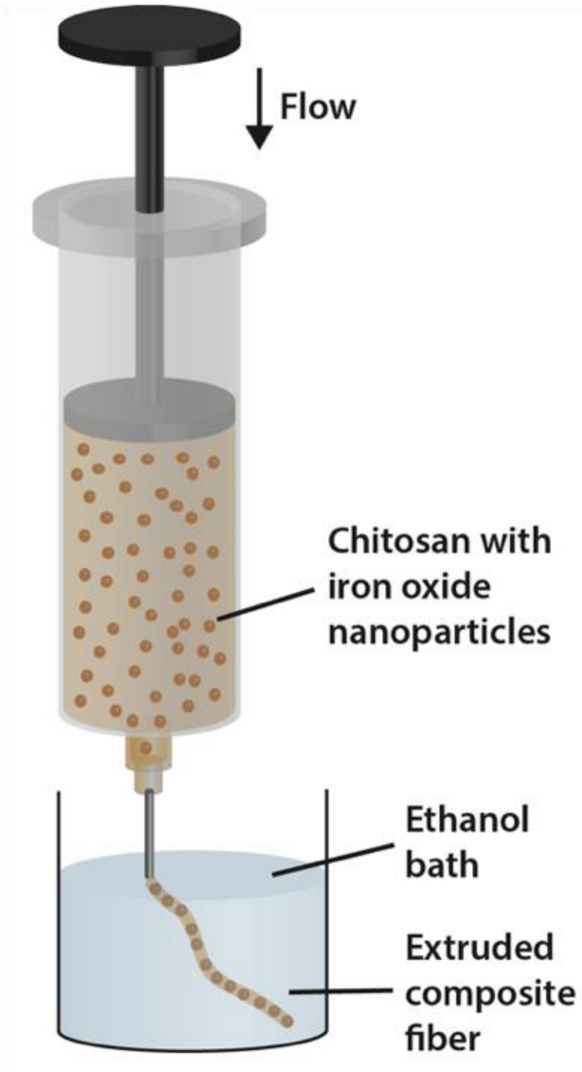

Figure S1: Schematic of the custom-built setup for wet-spinning of chitosan solutions blended with IOPs. Viscous feedstock solutions were extruded into an ethanol coagulation bath to facilitate fiber preparation.

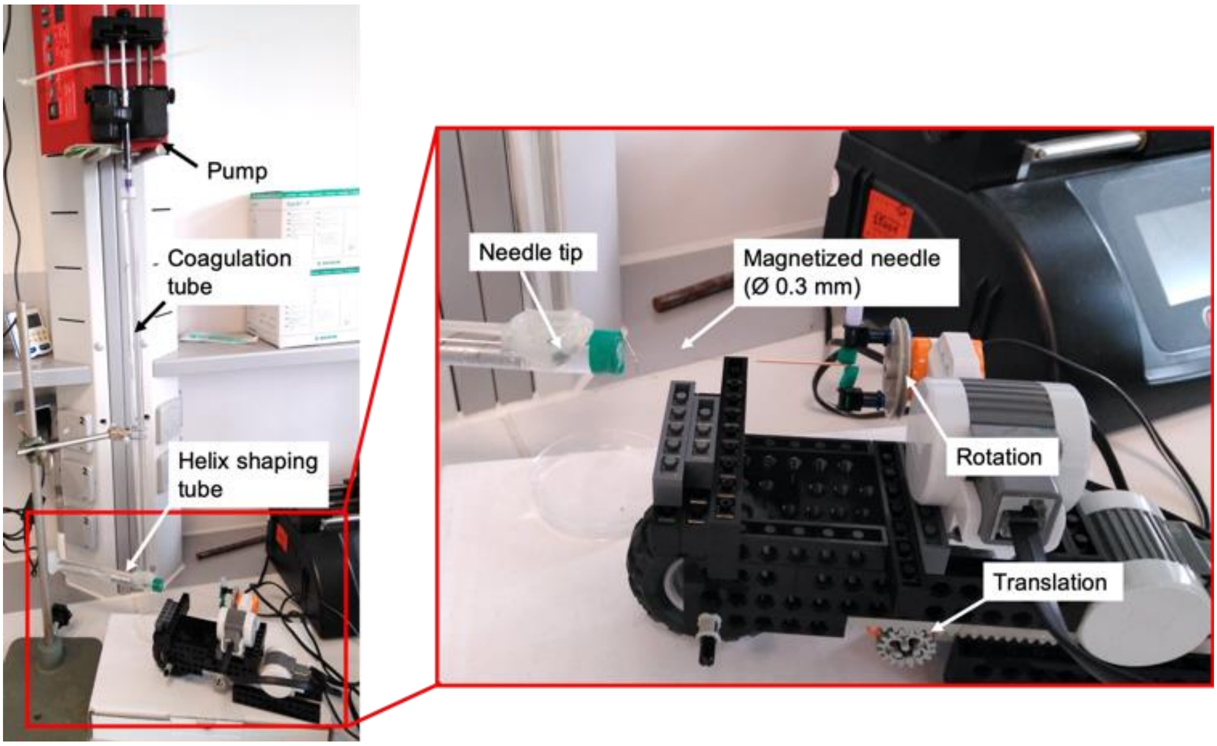

Figure S2: Custom-built setup for the wet-spinning process of helical microfibers. A solution of chitosan containing magnetic iron oxide nanoparticles is extruded into an ethanol coagulation bath. The emerging fibers are collected by a teflon-coated rotating needle with a stainless steel core, which simultaneously performs a translatory movement to achieve a helical fiber shape.

Table S1: Magnetic properties of the samples as determined with VSM.

VSM results	iron oxide concentration			
	1 mg·mL^{-1}	4 mg·mL^{-1}	7 mg·mL^{-1}	10 mg·mL^{-1}
saturation magnetization [T]	0.012	0.041	0.041	0.052
remanent magnetization [T]	2.99×10^{-4}	7.69×10^{-6}	7.69×10^{-6}	4.80×10^{-3}
coercive field [A·m^{-1}]	−150	−231	−231	−211
BH_{max} [MGsOe]	4.65×10^{-5}	3.16×10^{-4}	3.16×10^{-4}	3.15×10^{-4}

Table S2: Dynamic light scattering analysis of IOP dispersions in water.

pH	ZP [mV]	pH	Z-ave [nm]	number mean [nm]
10.9	−50.1	10.9	888	128
6.32	−26.4	6.4	684	103
6.52	−27.8	6.6	445	115
3.14	29.9	3.2	380	95
